# A Shift in Glycerolipid Metabolism Defines the Follicular Fluid of IVF Patients with Unexplained Infertility

**DOI:** 10.3390/biom10081135

**Published:** 2020-07-31

**Authors:** Albert Batushansky, Anish Zacharia, Alaa Shehadeh, Reut Bruck-Haimson, Daniel Saidemberg, Natalya M. Kogan, Chanchal Thomas Mannully, Shmuel Herzberg, Assaf Ben-Meir, Arieh Moussaieff

**Affiliations:** 1 The Institute for Drug Research, The Hebrew University of Jerusalem, Jerusalem 9112102, Israel; ala.shehadeh@mail.huji.ac.il (A.S.); reut.bruck@mail.huji.ac.il (R.B.-H.); daniel.saidemberg@mail.huji.ac.il (D.S.); natalya.kogan@mail.huji.ac.il (N.M.K.); chanchal.thomas@gmail.com (C.T.M.); 2 Aging and Metabolism Research Program, Oklahoma Medical Research Foundation, Oklahoma City, OK 73104, USA; 3 IVF Unit, Hebrew University Hadassah Medical Center, Jerusalem 9112001, Israel; herzberg3@gmail.com (S.H.); assaf.benmeir@gmail.com (A.B.-M.)

**Keywords:** follicular fluid, in vitro fertilization (IVF), lipidomics, triacylglycerols, monoacylglycerols, phospholipids, unexplained infertility

## Abstract

Follicular fluid (FF) constitutes the microenvironment of the developing oocyte. We recently characterized its lipid composition and found lipid signatures of positive pregnancy outcome after in vitro fertilization (IVF). In the current study, we aimed to test the hypothesis that unexplained female infertility is related to lipid metabolism, given the lipid signature of positive-outcome IVF patients we previously found. Assuming that FF samples from IVF patients with male factor infertility can represent a non-hindered metabolic microenvironment, we compared them to FF taken from women with unexplained infertility. FF from patients undergoing IVF was examined for its lipid composition. We found highly increased triacylglycerol levels, with a lower abundance of monoacylglycerols, phospholipids and sphingolipids in the FF of patients with unexplained infertility. The alterations in the lipid class accumulation were independent of the body mass index (BMI) and were altogether kept across the age groups. Potential lipid biomarkers for pregnancy outcomes showed a highly discriminative abundance in the FF of unexplained infertility patients. Lipid abundance distinguished IVF patients with unrecognized infertility and provided a potential means for the evaluation of female fertility.

## 1. Introduction

Infertility occurs in a 9% to 18% prevalence in the general population [1]. Infertility etiology includes ovulation dysfunction, mechanical factor (uterine cavity or blocked fallopian tubes) and male infertility (mainly abnormal semen analysis). In cases of infertility investigation without any abnormal result, an “unexplained infertility” is diagnosed in ~20–30% of infertile couples. In some of the cases, the “unexplained” reason can be further explored later during in vitro fertilization treatments and reveal abnormalities such as low fertilization rate or abnormal eggs. However, in most cases, the “unexplained” etiology for infertility remains obscure [2,3,4,5].

The oocyte quality and developmental competence are intrinsic to the development of a viable embryo. Follicular fluid (FF) is the liquid that surrounds the oocyte, forms its microenvironment and plays a key role in its development [1,2]. The oocyte microenvironment provides the necessary requirements for oocyte developmental competence, which is defined as the ability of the oocyte to complete meiosis and undergo fertilization, cleavage and fetal development to a healthy baby. These concerted processes require coordinated cellular metabolism with changing energy requirements [3].

Aging and the body mass index (BMI) are tightly associated with female fertility. Both factors have been suggested to influence the metabolic content of the FF and, in particular, the energy metabolism and lipid metabolism [4,5,6,7,8,9]. Surprisingly, while the mean number of mature oocytes was associated with fatty acid serum levels [10], BMI values did not associate with the fatty acid composition of the FF [11], leaving the mechanism for the association of maternal overweight to infertility thus far unknown [12,13,14,15]. It is likely, however, that the influence of systemic metabolism on female fertility involves changes in the FF, which are mediating maternal signals to the oocyte. In-line with this notion, the total lipid content of FF is increased in obese women, leading to lipotoxicity, impaired oocyte maturation and early embryonic loss [6,16,17,18]. Further to the total lipid content, changes in the lipid composition were previously associated with oocyte development. Triacylglycerol (TAGs) concentrations inversely correlated with the follicle size [19] and positively correlated to maternal BMI [6,18]. TAG accumulation in the FF were also correlated to the levels of adipokines and proinflammatory cytokines in FF [20], implying inflammatory processes in the FF that are caused by high TAG levels and may attenuate oocyte development. Several phosphocholines showed lower accumulation in poor ovarian responder patients [21]. Choline and phosphocholine—precursors of phosphatidylcholines, also accumulated in the FF of oocytes developed into early cleavage-stage embryos [22], whereas the total concentration of phospholipids (PLs) was implied to be inversely correlated with higher percentages of fertilized oocytes [23]. Recent studies have suggested a relationship between the concentration of vitamin D derivatives (a subgroup of cholesterol derivatives) and in vitro fertilization (IVF) outcome. However, these mostly focus on 25-hydroxyvitamin D only and offer conflicting evidence on its concentration and roles in the FF [24,25,26]. The FF levels of four sphingolipids (SL) species were also previously positively correlated with the oocyte cleavage rate [27]. 

We recently described the FF lipid composition in correlation with pregnancy outcome [28]. The lipid signature of FF that corresponded with a positive pregnancy outcome included a lower accumulation of TAGs, diacylglycerols (DAGs) and cholesteryl esters but a high accumulation of PLs, SLs and vitamin D derivatives. 

As a step towards revealing possible unknown factors causing female fertility, it would be important to characterize the microenvironment of the oocyte of infertile IVF patients with unexplained infertility. 

Here, we wanted to unveil the possible metabolic perturbations that relate to “unexplained” female infertility. Such metabolic alterations may shed new light on the etiology of female infertility of unknown background and provide potential biomarkers for it. Patient recruitment for a control group with normal, non-hindered follicle metabolism is a hurdle for such an endeavor. In the current study, FF from IVF patients with male-infertility backgrounds and no known maternal infertility factors (ruling out anovulation and mechanical factors) were selected and collected as a control group. Notably, as there is no examination to prove female fertility besides conceiving, the division to experimental groups is inherently challenging. Moreover, although 20% of infertile couples suffer from combined male and female infertility, in women without any abnormal fertility factor, the chance for combined infertility is low. Given the above considerations, we compared the lipid composition of FF from patients of this control group to that of FF from IVF patients with no known cause for infertility and reasoned that such a comparison should unveil unknown links between thus far unexplained infertility and follicle lipid metabolism, and, hopefully, may also provide new clinical indications for female infertility. 

## 2. Materials and Methods 

### 2.1. Study Population

Patients undergoing fertility treatment were recruited at the IVF Unit of the Hebrew University Hadassah Medical Center. The Institutional Review Board of Hadassah Medical Organization approved the study (decision number 0207-15-HMO), and each patient signed a consent form before oocyte retrieval. Patients included in the study were either with male infertility background (“male factor”) or negative outcome patients with unknown infertility background (“unexplained background”). The patients with unexplained infertility reported in the current study are a part of a cohort used for a previously reported study [28]. Exclusion criteria included no embryo transfer. Patients underwent controlled ovarian hyper-stimulation by the GnRH antagonist protocol or short GnRH agonist protocol. The ovarian response was assessed by transvaginal ultrasound and estradiol (E2) levels every 2 to 3 days. Human chorionic gonadotropin, the GnRH agonist or both were administered to induce final oocyte maturation 36 h before oocyte retrieval. Oocyte retrieval was performed under general anesthesia using transvaginal aspiration with 16–17-gauge needles under ultrasonography guidance. Oocytes were extracted by an embryologist, and the residual FF was pooled and spun down to collect FF and discard blood and granulosa cells. Fertilization was accomplished by IVF or intracytoplasmic sperm injection. The embryos were cultured in individual wells on a plate in a time-lapse incubator (EmbryoScope; Vitrolife, Gothenburg, Sweden). Embryo transfer was done after 3–6 d according to physician preference and the embryo morphologic grading. A human chorionic gonadotropin (HCG) pregnancy test was carried out 2 weeks after embryo transfer.

### 2.2. Materials and Reagents

Solvents were purchased from JT Baker (Phillipsburg, NJ, USA), except for isopropanol (Chemsolute; Th. Geyer, Renningen, Germany). All solvents but chloroform (HPLC grade) were liquid chromatography mass spectrometry (LC-MS) grade. Formic acid was purchased from Tokyo Chemical Industry (Tokyo, Japan). Leucine enkephaline-TOF-G2 XS Standard Kit was purchased from Waters (Milford, MA, USA), and sodium formate (98%) and ammonium fluoride (LC-MS) were purchased from Fluka (Buchs, Switzerland).

### 2.3. Sample Preparation for LC-MS Analysis

Freshly collected samples were immediately centrifuged at 770 g (10 min, 4 °C) to separate FF from cells and debris. Supernatant was transferred to new tubes, snap-frozen in liquid nitrogen and stored at −80 °C until analysis. Lipid extraction was performed as previously described [28]. Briefly, an optimized Bligh and Dyer biphasic extraction was used to extract lipids. Lipid extracts were transferred to new tubes and dried in a SpeedVac concentrator (Thermo Fisher Scientific, Waltham, MA, USA). Samples were then resuspended in 200-μL acetonitrile 95%, 0.1% formic acid, filtered through a 0.22-μm polytetrafluoroethylene (PTFE) membrane and transferred to LC-MS vials.

### 2.4. Ultra-HPLC Quadrupole Time-of-Flight MS Analysis

Waters Acquity UPLC H-Class equipped with a photodiode array detector, together with Waters high-resolution, high-mass accuracy Xevo X2-XS Q-ToF, were employed for lipid analysis. Following previous analyses [28], electrospray ionization (ESI) was applied in positive mode (ES+). Chromatographic separation was carried out using a UPLC CSH C18 column (100 × 2.1 mm, 1.7 μm; Waters). Mobile phase composition, gradient program and MS parameters were the same as previously reported [28]. In addition to FF samples, blanks and quality control (QC) samples (herein) were generated and went through all sample preparation (see above) to be included in the LC-MS analysis. Blanks consisted of solvents used for sample preparation. Two QC samples were introduced. A QC sample was prepared by collecting and pooling 5-µL aliquots of all FF samples to best represent the matrix studied. A second QC set consisted of 9 external lipid analytical standards: arachidonic acid 5 μM; N-hexanoyl-D-sphingosine 10 μM; stearoyl-SN-glycero-3-phosphocholine 10 μM; 24:0 C24 ceramide-1-phosphate (d18:1/24:0) 10 μM and a triglyceride mix (a mix of 5 standards): triacetin (C2:0), tributyrin (C4:0), tricaproin (C6:0), tricaprylin (C8:0) and tricaprin (C10:0).

### 2.5. Data Analysis

Data acquisition and visualization was performed using MassLynx 4.1 (Waters). Masses of features that eluted earlier than 1.0 min were excluded from the analysis, and a minimum mass intensity cutoff was set to 100 m/z. Potential artificial features originated during sample preparation were excluded from the dataset by excluding features with the lowest abundance in the blank samples and omitting masses with lower intensity than 100-fold change (FC) from the highest intensity in the blank. Mass spectra were aligned and subjected to identification and quantification of FF-derived features using Progenesis QI (Nonlinear Dynamics, Newcastle, UK). Exact mass (mass accuracy < 5 PPM), retention time, isotope and fragmentation patterns were used for identification against 18 Progenesis Q-compatible metabolite libraries. The identification of lipid species was further validated by manual comparison of their mass fragments to theoretical and literature fragments. Data was normalized to total intensity (area). Missing values (representing values below the detectability threshold) were imputed by half of the smallest detected value for the specific feature. Following annotation and Log-transformation, 401 features were subjected to the orthogonal partial least-squares discriminant analysis (OPLS-DA) using the ropls package for R [29]. Correlation analysis of the annotated features was performed by computing Pearson’s pairwise correlation coefficients, as previously described [30]. The output provided two separate correlation matrices (one for each group). Strong (r > |± 0.7|) and significant (*p* < 0.001) correlations were visualized as heat-maps using the corrplot package for R [31]. 

## 3. Results

### 3.1. Collection of FF Samples from IVF Patients with Unexplained Infertility or Male Factor Background

Following our previous work that demonstrated a differential lipid composition of FF from IVF patients with positive pregnancy outcomes, we reasoned that currently unexplained infertility may be related to femalelipid metabolism. To test a possible link between follicular lipid metabolism and unexplained infertility, we collected 30 FF samples of unexplained background patients. Under the assumption that FF from IVF patients with male infertility background and no known female infertility factors should have normal (similar to that of fertile women) lipid compositions, we collected FF samples from 30 male factor backgrounds as control samples. FF from both groups was subjected to a lipidomics analysis. Clinical characteristics of the cohort patients are presented as Table 1.

### 3.2. A Differential Lipid Composition of the FF of IVF Patients with Unexplained Infertility 

**Figure 1:**
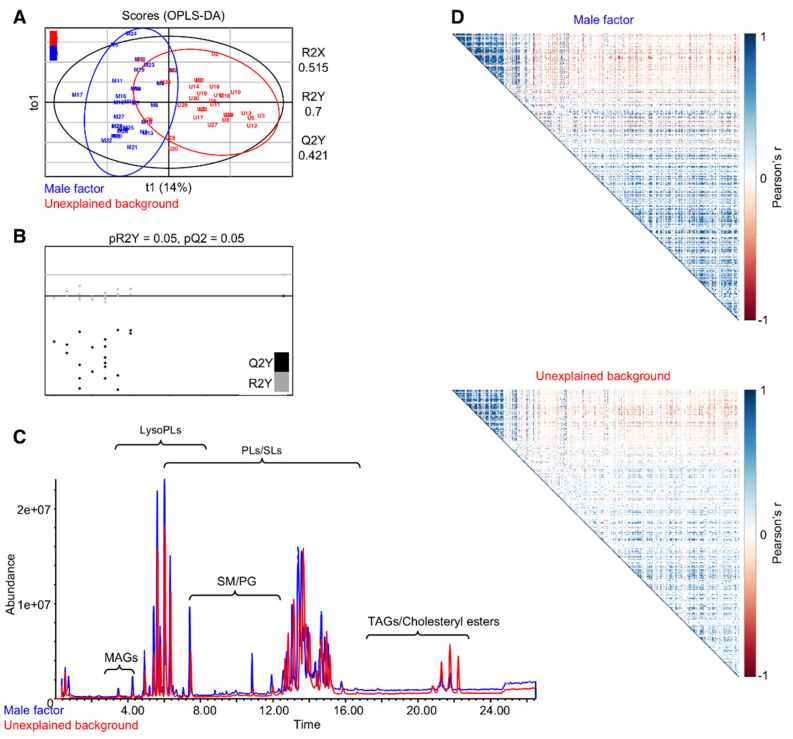
Follicular fluids of unexplained infertility patients show a differential lipid profile when compared to follicular fluids from patients with a male infertility factor. Annotated features (401) subjected to orthogonal partial least-squares discriminant analysis (OPLS-DA) demonstrate an alteration in the lipid profile of unexplained infertility patients (**A**), validated by a permutation test (**B**). Representative chromatograms show differences in the relative abundances of lipids across the chromatogram (**C**). Pearson’s correlation matrices between 401 annotated features. Significant correlations (*p* < 0.001) were visualized as heat maps (**D**). No clustering was applied, to enable a direct comparison between the two matrices. PLs: phospholipids, SM: sphingomyelins and TAGs: triacylglycerols.

We detected 9953 mass features in the FF sample set. Following the exclusion of low abundant features (intensity < 100), features that elute between 0–1 min, and features that are present in the blank control samples (see Materials and Methods section), 1571 features were assigned as FF-derived features. Of these, 401 features were identified. Following our previous analyses that suggest that vitamin D derivatives show a higher accumulation in the FF of positive-outcome IVF patients, we further carried out a targeted analysis of features with vitamin D mass fragments, and seven features were annotated as derivatives of Vitamin D. An OPLS-DA model developed for the annotated features of the untargeted analysis suggested a trend for separation (Figure 1A), with an acceptable fit (R^2^Y = 0.7) but relatively low prediction (Q^2^Y = 0.42), raising concern as to the predictive performance of the model. We therefore assessed the predictive performance of the model by a permutation test (20 permutations; Figure 1B), which suggested that the model developed for separation of the lipid composition of the two groups was predictive. Supporting the separation of the lipid composition of FF of the two groups, the corresponding chromatograms demonstrated differences in the relative abundance of features across several retention time frames and, in particular, in the typical retention time window of TAGs and cholesteryl esters (Figure 1C). Multiple studies have demonstrated that an evaluation of the correlations between variables can emphasize differences in the systemic response even when individual quantitative differences are minor [32,33,34,35]. We generated correlation matrices for significant correlations (*p* < 0.001) between the 401 identified features. To enable a comparison between the two matrices, the order of features was kept (i.e., no clustering of features was carried out), and matrices were visualized as heat maps (Figure 1D). Notably, the pattern of correlation between the FF of unexplained background and male factor patients is closely related. However, the number of correlations (both positive and negative) and their intensities is considerably higher in the male factor correlation matrix. For further visualization of the different patterns of correlations between the lipid species in the two groups, the clustered (hierarchical algorithm) correlation heat maps are presented as Figure S1. 

### 3.3. Glycerolipid Metabolism Shifts From Monoacylglycerols to Triacylglycerols

**Figure 2:**
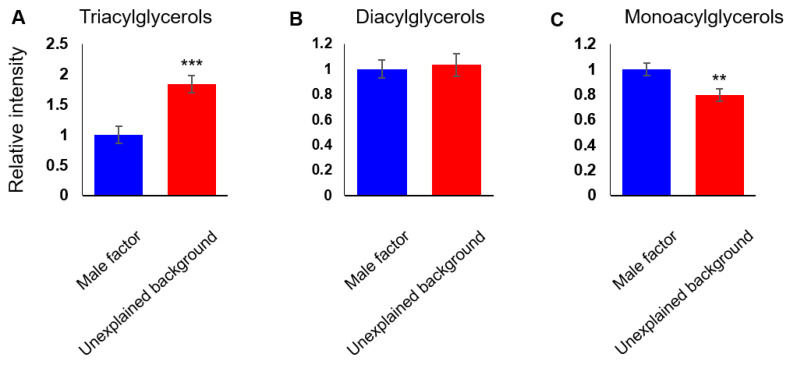
A shift in glycerolipid metabolism is seen in unexplained infertility patients. The cumulative abundance of lipid species was measured for glycerolipid subclasses: Triacylglycerols (**A**), diacylglycerols (**B**) and monoacylglycerols (**C**). Bars are means ± SEM, *n* = 30 per group. ** (*p* < 0.01) and *** (*p* < 0.001) calculated by Student’s *t*-test.

**Figure 3:**
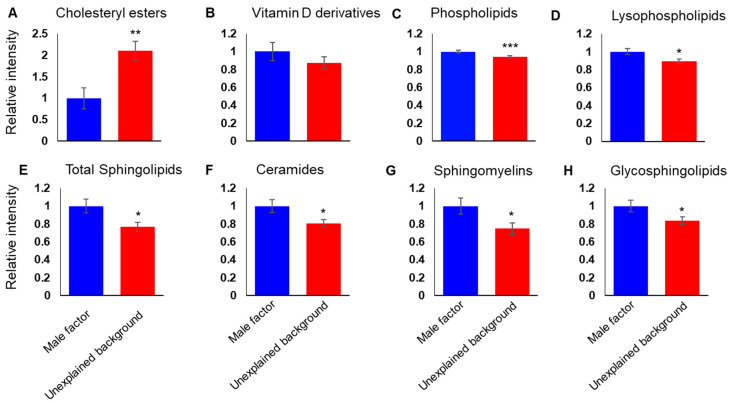
Further lipid classes show a differential relative abundance in unexplained infertility patients consistent with the shift in the glycerolipid metabolism. The cumulative abundance of the lipid species was measured for different lipid classes: cholesteryl esters (**A**), vitamin D derivatives (**B**) phospholipids (**C**), lysophospholipids (**D**) total sphingolipids (**E**), ceramides (**F**), sphingomyelins (**G**) and glycosylated sphingolipids (**H**). Bars are means ± SEM. * (*p* < 0.05), ** (*p* < 0.01) and *** (*p* < 0.001) (Student’s *t*-test).

Taken together, our data suggested that the lipid composition of FF from unexplained background patients is different from that of the male factor group, and that the differences are across several lipid classes, with both positive and negative correlations between the lipid features. For a detailed understanding of the differences in the FF lipid composition of IVF patients of different backgrounds, we studied the relative abundance of different lipid classes. TAGs are the main constituents of body fat and are present in the blood and FF. We found higher levels of TAGs (1.8-fold change, degrees of freedom (df) = 59, *p* < 0.01; Figure 2A) in the FF of unexplained background patients. We saw no difference in the abundance of diacylglycerols (DAGs; Figure 2B), and the abundance of monoglycerols (MAGs) was lower in the FF of unexplained background patients (0.8-fold change and *p* < 0.01; Figure 2C). Altogether, a distinct shift was seen in glycerolipid metabolism. Another notable class of lipids with considerably higher levels in unexplained background FF is cholesteryl esters (2.1-fold change and *p* < 0.01; Figure 3A). While the abundance of cholesteryl esters was higher in unexplained background FF, the abundance of 7-dehydrocholesterol-derived lipids (vitamin D derivatives) did not change significantly (Figure 3B). Contrary to the higher abundance of TAGs and cholesteryl esters in the FF of unexplained background patients, we saw a lower accumulation of PLs (0.9-fold change and *p* < 0.001; Figure 3C) and lysoPLs (phospholipids with a single acyl chain) (0.9-fold change and *p* < 0.05; Figure 3D). The total abundance of SLs was also lower in the FF of unexplained background patients (0.8-fold change and *p* < 0.05; Figure 3E), as seen in all subclasses: ceramides (0.8-fold change, *p* < 0.05; Figure 3F), sphingomyelins (SMs; 0.8-fold change and *p* < 0.05; Figure 3G) and glycosphingolipids (0.8-fold change, *p* < 0.05; Figure 3H).

### 3.4. Potential Lipid Biomarkers for Pregnancy Outcome Show Discriminant Accumulation in the FF of IVF Patients with Unexplained Infertility

We previously found 32 potential lipid biomarkers in the FF of positive-outcome IVF patients and evaluated their predictive ability for the outcome of pregnancy by testing six of them in a receiver operating characteristic curve (ROC) analysis. This analysis yielded an 86% area under curve [28]. To test the potential lipid biomarkers for differential accumulation in the FF of IVF patients with an unknown cause of infertility, we compared their abundance in the FF of unexplained background vs. male factor patients. Of the 32 potential biomarkers, two had 0.05 ≤ q ≤ 0.1 and 30 had q-values under 0.05 (Table 2), indicating their potential as markers of female-related infertility. 

### 3.5. The Lipid Signature of Patients with Unexplained Infertility is not Age- or BMI-Dependent

**Figure 4:**
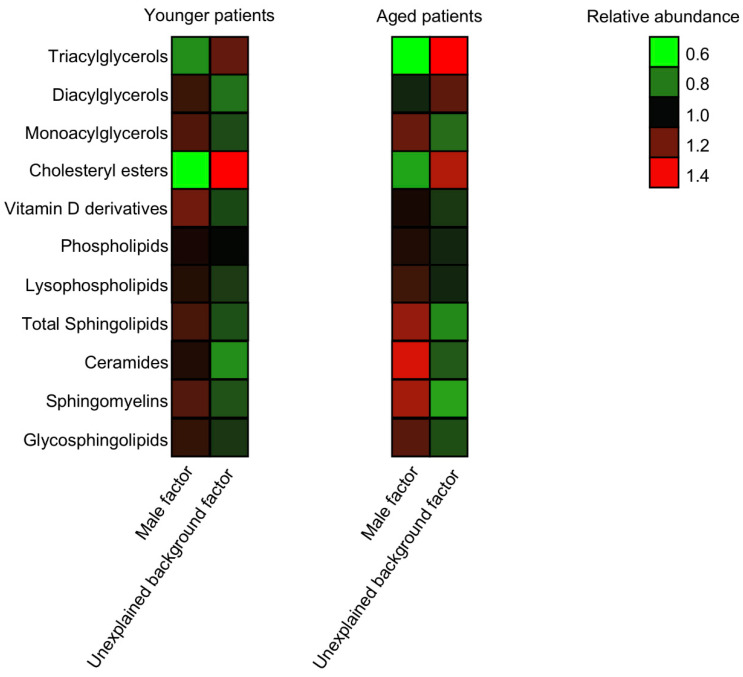
The alterations in the lipid composition are across age groups. The cumulative abundance of the lipid species was measured for different lipid classes in younger (age < 32) and aged (age > 39) patients. *n* male = 19 and *n* unexplained = 6 in the younger group, and *n* male = 5 and *n* unexplained = 19 in the aged group.

Given the well-established association between BMI and female fertility, and the differences found in BMI values between IVF patients of the two groups (Table 1), we studied the correlations between BMI and the abundance of the lipid classes in the samples under study using a Pearson’s correlation analysis. Surprisingly, the correlation values of all lipid classes were below 0.5, suggesting very weak correlations (data not shown). Age is a critical factor affecting healthy female fertility [36]. To decipher age-related alterations in the FF lipid composition of unexplained background IVF patients, we dissected our cohort into two groups of ages: under 32 (“younger”, *n* = 25) and over 39 (“aged”, *n* = 24). The metabolic alterations were altogether consistent across the age groups, with almost all trends kept. The only class of lipids that showed a different trend in the two age groups was DAGs, with a nonsignificant higher accumulation in the FF of younger male factor FFs (Figure 4).

## 4. Discussion

While increased body fat is well-associated with reduced female fertility, the roles played by lipid metabolism in the development of the oocyte are unclear. The mechanism by which fat-related signals are transduced to affect the developing oocyte is also still obscure [12,13,14,15]. 

In this study, we aimed to unveil possible relationships between unexplained infertility and the metabolic microenvironment of the developing oocyte. Given our previous work that suggests an association of the FF lipid composition to the outcome of pregnancy, we focused on the lipid fraction of small molecules, i.e., lipidome. A limitation that should be noted in the study design is the possibility for combined male and female infertility. However, in women without any abnormal fertility factors, the chance for combined infertility is low. Given this limitation, our multivariate analyses revealed differences between the two groups examined, and notable associations between the abundances of the lipid species. Although interpretation of the correlation between metabolites is not straightforward, this result reflects differences in the infrastructure linking multiple biochemical reactions and their regulatory processes within each group [37]. Taken together with the correlation analysis, the separation in our OPLS-DA model, which was confirmed by a permutation test suggests a link between unexplained infertility and the lipid composition of the microenvironment of the oocyte. Our downstream analyses of lipid class accumulation primarily point to a shift from the accumulation of MAGs and PLs to TAGs. While the control of TAG synthesis varies from tissue to tissue, at a low fatty acid availability and a low rate of formation of DAGs, the major flux from DAGs is directed to the synthesis of phosphatidylcholine and phosphatidylethanolamine [38], providing a possible explanation for the shift seen in our analyses. In bovine, TAGs are a primary energy source in the developing oocyte [39]. Nevertheless, a high TAG content in the FF was correlated to a reduced follicle size [19] and an increased expression of adipokines and proinflammatory cytokines in the FF [20]. It is therefore likely that local concentrations of TAGs are important for the development of the oocyte but are harmful in higher concentrations. Given the reported higher TAG content in bovine oocytes that grow in fetal calf serum-supplemented medium [40], these results bear practical implications. TAGs are metabolized by lipases that have been localized to cumulus cells, as well as oocytes [41]. It is also reported that human granulosa-lutein cells assemble and secrete in vitro-native VLDL particles with high TAG contents [42]. The lack of correlation between the abundance of TAGs, as well as other lipid classes, to patient BMI in our cohort is surprising, especially given previous reports on the association between BMI and the FF metabolic composition [6]. Together with the above previous reports, it may imply an alteration in the local TAG metabolism of unexplained background patients. Such an alteration is likely to result from a local differential expression of enzymes in the glycerolipid pathway. It would be important to evaluate the expression of enzymes responsible for TAG synthesis and of the corresponding lipases in follicular cells. The non-BMI-related changes in the FF lipid composition that are linked to unexplained infertility underscore the importance of a better understanding of the lipid metabolism in the microenvironment of the oocyte. Nevertheless, if TAG concentrations in the FF may be correlated to their concentration in the blood, our results may provide a straight forward, and more reliable and relevant means for the evaluation of the metabolic state of the IVF patient than the currently used BMI. 

Age is a major factor affecting healthy female fertility, with a fertility decline starting already around 25–30 years of age, and the median age at last birth is 40–41 years in most studied populations experiencing natural fertility [36]. When we compared the lipid composition of the male factor and unexplained background samples within each age group, we found that the metabolic signatures of unexplained infertility are altogether consistent across the age groups, with almost all trends kept. The association of unexplained infertility across the age groups is in-line with the association of positive outcomes of pregnancy previously demonstrated by us. It should, however, be noted that the age groups are imbalanced in their sample sizes, due to the higher availability of samples from unexplained infertility cases from aged IVF patients.

The lipid signatures of male factor FF samples closely resemble the previously reported signatures of FFs from positive-outcome patients [28]. The shift in the accumulation of glycerolipids towards a lower abundance of TAGs is more evident in the current study, potentially due to a more discriminant glycerolipid metabolism in the FF of male factor patients, which should represent a non-hindered microenvironment. The similarity between the metabolic signatures of IVF patients with negative outcome to the signatures that distinguish the FF of unexplained infertility patients from male factor patients corroborates the notion that the association between the FF lipid composition and pregnancy outcome is due to unknown lipid-related factors in female fertility. 

The distinct accumulation of the potential lipid biomarkers in the unexplained background when compared to the male factor FF supports their potential predictive value for female infertility.

The lipid metabolic signatures and, especially, the shift we found in the glycerolipid metabolism are likely to represent an unfavorable lipid microenvironment for the oocyte. Such alterations in the metabolic microenvironment may be partially responsible for unexplained cases of female infertility. The mechanistic details of this relationship require further study. Such mechanisms may involve high local concentrations of fatty acids and a corresponding stress, resulting in thus far unexplained infertility. 

## 5. Conclusions

Our study reveals a distinct lipid composition of the FF of unexplained infertility IVF patients. Our analyses suggest that unexplained female infertility is linked to a metabolic shift from MAGs to TAGs. The metabolic shift from MAGs, PLs and SLs to TAGs may shed new light on the etiology of female infertility. Given the differences in the abundance of potential lipid biomarkers, their concentrations may help to identify the male/female source of infertility. 

## Figures and Tables

**Table 1:** Clinical characteristics of the cohort patients. BMI: body mass index.

Characteristics	Male Factor Infertility (*n* = 30)	Unexplained Infertility (*n* = 30)	*p*-Value
Age (years)	32 ± 5.2	38 ± 5.2	<0.001
BMI (kg/m^2^)	23.5 ± 3.6	27.2 ± 6.9	0.02
Previous Pregnancies	0.83 ± 1.1	0.7 ± 1.1	0.65
Previous Deliveries	0.53 ± 1.02	0.13 ± 0.34	0.05
Previous Miscarriages	0.27 ± 0.51	0.43 ± 0.88	0.38
No. of previous IVF cycles	2 ± 1.6	1.9 ± 1.1	0.78
No. of retrieved oocytes	11.83 ± 8.39	6.5 ± 5.7	0.006
Maximal serum estradiol (pM)	7434 ± 4109	4828 ± 2989	0.009
Sperm Parameters:			
Volume	2.8 ± 1.3	2.8 ± 0.9	0.94
Concentration	6.4 ± 9.9	53.2 ± 31.1	<0.01
Motility	16.6 ± 16.1	32.8 ± 19.7	<0.01
Morphology	3.2 ± 4.9	4.7 ± 3.6	0.56

**Table 2:** Potential lipid biomarkers for pregnancy [28] show a unique abundance in the follicular fluid of in vitro fertilization (IVF) patients with unexplained infertility backgrounds. The identification of the lipid species was validated by a comparison of their mass fragments to theoretical and literature fragments following tandem MS/MS transitions.

Putative Identification	Molecular Ion/Precursor Ion	Empirical Formula (Molecular Ion)	log2 (FC)	q-Value *
TG(16:1/18:0/20:0)	906.84 (M+NH4)	C_57_H_108_O_6_	2.04	0.00002
TG(14:1/16:0/20:0)	850.78 (M+NH4)	C_53_H_100_O_6_	2.04	0.00003
TG(16:0/16:0/16:1) **	822.75 (M+NH4)	C_51_H_96_O_6_	1.96	0.00003
TG(18:1/14:0/22:1)	904.82 (M+NH4)	C_57_H_106_O_6_	1.47	0.00004
TG(18:0/16:0/18:0)	904.83 (M+ACN+H)	C_55_H_106_O_6_	1.66	0.00004
TG(14:1/19:0/22:1)	918.84 (M+NH4)	C_58_H_108_O_6_	1.85	0.00004
TG(14:1/20:0/21:0)	920.86 (M+NH4)	C_58_H_110_O_6_	2.56	0.00008
TG(18:1/16:0/18:0)	878.81 (M+NH4)	C_55_H_104_O_6_	2.11	0.00015
TG(15:1/24:1/18:2) **	1008.89 (M+IsoProp+Na+H)	C_60_H_108_O_6_	1.53	0.00027
TG(16:0/16:1/16:1)	820.73 (M+NH4)	C_51_H_94_O_6_	1.49	0.00041
LysoPC(18:2)	520.61 (M+H)	C_26_H_50_NO_7_P	−2.16	0.00181
TG(14:0/16:0/16:1)	794.72 (M+NH4)	C_49_H_92_O_6_	1.76	0.00185
PC(P-16:0/20:2)	1562.18 (2M+Na)	C_44_H_84_NO_7_P	−1.44	0.00241
Unidentified	959.13		1.71	0.00241
Unidentified	1044.7055		−2.14	0.01089
Unidentified	1005.69		−2.12	0.01388
TG(16:1/18:1/18:1)	876.80(M+NH4)	C_53_H_102_O_6_	2.18	0.01467
Unidentified	524.907		−2.30	0.01625
Unidentified	783.9095		−2.02	0.01625
Unidentified	759.4597		−2.14	0.01625
Unidentified	1036.93		1.84	0.01625
LysoPC(18:0)	524.371 (M+H)	C_26_H_54_NO_7_P	−2.24	0.01774
Lyso PC(18:1)	1043.702 (2M+H)	C_26_H_52_NO_7_P	−2.20	0.02053
LysoPC(18:1)	522.35 (M+H)	C_26_H_52_NO_7_P	−2.24	0.02191
Unidentified	783.4730		−2.10	0.02191
Unidentified	1056.89		1.28	0.02226
TG(14:0/18:3/16:0)	818.72 (M+NH4)	C_51_H_92_O_6_	0.82	0.02600
TG(18:0/24:0/20:4)	1058.9 (M+ACN+Na)	C_65_H_118_O_6_	1.19	0.02882
TG(20:0/20:3/22:0)	1060.92 (M+ACN+Na)	C_65_H_120_O_6_	1.45	0.03621
TG(20:3/20:1/22:0) **	1058.90 (M+ACN+Na)	C_65_H_118_O_6_	1.13	0.04079
SM(d18:1/16:0) **	1406.141 (2M+H)	C_39_H_79_N_2_O_6_P	−1.31	0.05886
TG(20:0/22:3/22:2)	1084.92 (M+ACN+Na)	C_67_H_120_O_6_	1.16	0.08095

* Multiple testing correction was performed using the false discovery rate (FDR) and the Benjamini & Hochberg method, adjusted to 32 potential biomarkers. ** Fragments taken from the MS^E^ analysis. FC: fold change.

## Supplementary Materials

The following are available online at https://www.mdpi.com/2218-273X/10/8/1135/s1, Figure S1: Hierarchically clustered heat-map visualization of the Pearson’s correlation matrices between 401 annotated features for Male factor (top) and Unexplained background (bottom).
