# Supplementary material for: A Shift in Glycerolipid Metabolism Defines the Follicular Fluid of IVF Patients with Unexplained Infertility
Source: Biomolecules. 2020 Jul 31;10(8):1135. doi: 10.3390/biom10081135 (PMC7465802; doi:10.3390/biom10081135)
Supplement: Supplementary file 1 [file biomolecules-10-01135-s001.pdf]

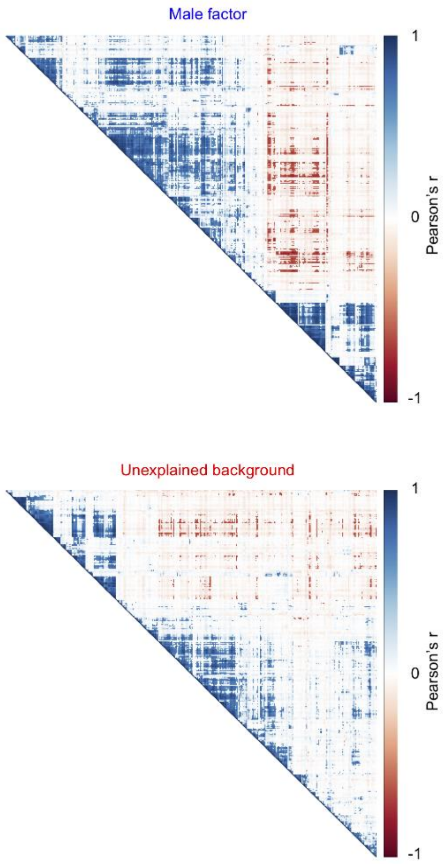

Supplementary Figure 1: Hierarchically clustered heat-map visualization of the Pearson's correlation matrices between 401 annotated features for Male factor (top) and Unexplained background (bottom). Only significant correlations ($p < 0.001$) were plotted.
